# Clinical outcomes of external fenestration TEVAR in Stanford type B aortic dissection involving the left subclavian artery: a retrospective study

**DOI:** 10.3389/fcvm.2026.1865765

**Published:** 2026-09-03

**Authors:** Chunshui Liang, Hong Liu, Xiaobo Peng, Tianbo Li, Chencheng Liu, Yong Wang, Yingbin Xiao, Ruiyan Ma

**Affiliations:** Department of Cardiovascular Surgery, Xinqiao Hospital, The Army Medical University, Chongqing, China

**Keywords:** aortic dissection, computational fluid dynamics, external fenestration, hemodynamics, left subclavian artery, thoracic endovascular aortic repair

## Abstract

**Objective:**

This study assessed the clinical efficacy and hemodynamic effects of external fenestration thoracic endovascular aortic repair (TEVAR) in patients with type B aortic dissection (TBAD) involving the left subclavian artery (LSA).

**Methods:**

A retrospective analysis of 110 patients who underwent this procedure between 2020 and 2024 was conducted, evaluating procedural success, complications, and follow-up outcomes. Hemodynamic changes were analyzed in 1 patient using computational fluid dynamics (CFD) based on pre- and postoperative CT angiography (CTA).

**Results:**

Technical success was achieved in all cases. Complications included 3 cases of type I endoleak (2.7%), 1 type II endoleak (0.9%), 3 type IV endoleak (2.7%), 1 LSA stenosis (0.9%), and 2 deaths (1.8%). At a 5.7-month follow-up, 92.7% of patients showed complete false lumen thrombosis. There were occurrences of type I endoleak (2.7%), type II endoleak (1.8%), type IV endoleak (2.7%), LSA occlusion (2.7%), and LSA stenosis (2.7%). CFD analysis in the representative case demonstrated restoration of physiological flow patterns, including normalized streamlines in the LSA and stabilized wall shear stress in the stented segment.

**Conclusion:**

External fenestration TEVAR demonstrates favorable clinical outcomes and hemodynamic stability in TBAD involving the LSA, effectively promoting false lumen thrombosis and aortic remodeling while preserving LSA perfusion. Our findings suggest that complete entry coverage with an adequate proximal landing zone (≥15 mm when anatomically feasible) may be important for achieving false lumen depressurization, although this observation requires further validation. Further prospective studies are warranted to validate these findings.

## 1 Introduction

Aortic dissection (AD) is a severe aortic condition that can cause rupture and organ malperfusion (e.g., myocardial infarction, cerebral infarction, renal failure) (1). Type B aortic dissection (TBAD), a subtype of AD, involves a tear in the aortic wall beyond the left subclavian artery and poses a serious threat (2). Thoracic endovascular aortic repair (TEVAR) is the standard treatment for most TBAD cases due to its minimal invasiveness and effectiveness, as demonstrated since its first use in 1999 (3–5). However, TEVAR requires sufficient healthy proximal landing zones. It cannot be used if the distance between the primary tear and the left subclavian artery is less than 15 mm, or if dissection or hematoma extends beyond the artery, even with a greater tear-to-artery distance. Covering the LSA with a stent graft is the easiest way to extend the proximal landing zone but increases the risks of left upper limb ischemia, subclavian steal syndrome, vertebral artery-type cerebral ischemia, and paraplegia (6, 7). Alternative methods like the chimney technique and hybrid surgery aim to maintain LSA perfusion while extending the landing zone, but the chimney technique has high risks of endoleak or branch occlusion (8), and hybrid surgery involves cervical incisions with potential complications (9). In contrast, external fenestration TEVAR is a minimally invasive approach that effectively preserves LSA patency and extends the landing zone with fewer complications and quicker recovery (10), although its hemodynamic effects are not well understood. At present, there are few studies with large sample sizes and involving hemodynamic data. Our study explores the clinical efficacy and hemodynamic impact of extra-anatomic fenestration in LSA-involved AD. In this study, we integrate patient-specific computational fluid dynamics (CFD) modeling with clinical data to assess aortic remodeling, branch perfusion, and hemodynamic stability, thereby providing insights for optimizing endovascular strategies in the management of complex TBAD.

## 2 Methods

### 2.1 Study population

This retrospective, non-randomized study was conducted at a high-volume, single-center institution and involved a review of 110 patients with TBAD involving the LSA, who were treated with external fenestration TEVAR between 2020 and 2024. Exclusion criteria included type A aortic dissection (TAAD), TBAD not involving the LSA, aortic rupture, concomitant cardiac and vascular diseases requiring surgical intervention, and renal failure characterized by serum creatinine levels ≥444 μmol/L or a glomerular filtration rate ≤15 mL/min. Among these, one representative case with high-quality pre- and post-operative ECG-gated CTA datasets (slice thickness ≤ 0.75 mm) and complete hemodynamic recordings were selected for CFD analysis.

### 2.2 Data variables and definitions

#### 2.2.1 Baseline characteristics

Baseline demographic and clinical data were collected from Xinqiao Hospital's Cardiovascular Surgery Department, affiliated with the Army Medical University. Aortic dissection was classified as acute (within 14 days of symptoms), subacute (15–90 days), or chronic (over 90 days). Chronic lung disease included chronic obstructive pulmonary disease (COPD) or pulmonary fibrosis requiring treatment, and kidney damage was defined as GFR <90 mL/min. All patients received the Medtronic Valiant™ Captivia™ thoracic stent graft system (Medtronic Inc., Santa Rosa, CA, USA), a device consisting of a woven polyester graft sutured to a self-expanding nitinol wire frame. The diameter of the custom-made external fenestration created on the graft was standardized to 8–10 mm, based on preoperative LSA diameter measurements.

#### 2.2.2 Statistical analysis

Numerical data were expressed as means and standard deviations, while categorical data were shown as counts and percentages. Categorical variables were analyzed using the Chi-square or Fisher's exact test, and continuous data with the independent samples t-test. Analyses were conducted using IBM SPSS Statistics version 27.

### 2.3 Device description

All patients enrolled in the study were treated using the Medtronic Valiant™ Captivia™ device. The details of the device have been described (11).

The external fenestration procedure was performed intraoperatively as follows: (1) Based on preoperative CTA measurements, the optimal fenestration position on the stent graft was determined to align with the left subclavian artery (LSA) ostium. (2) Under sterile conditions, the stent graft was partially deployed on a back-table, and a circular fenestration (8–10 mm in diameter, matching the LSA size) was created using a standard surgical blade or electrocautery device. (3) The graft was then carefully re-sheathed into the delivery system, ensuring that the fenestration remained correctly oriented relative to the delivery system's rotational markers. (4) During the endovascular procedure, the fenestrated stent graft was deployed at the targeted zone (zone 2 or proximal zone 3) with the fenestration precisely aligned with the LSA ostium, confirmed by immediate digital subtraction angiography. This technique preserves antegrade LSA perfusion without the need for supra-aortic bypass or chimney grafts.

### 2.4 Image processing and geometry reconstruction (representative CFD case)

Patient-specific geometric models of aortic arch dissection before and after TEVAR were reconstructed based on clinical contrast-enhanced computed tomography angiography (CTA) DICOM datasets, following the standardized vascular image processing workflow reported in previous aortic dissection CFD studies. All original CTA images possessed high spatial resolution, which enabled clear differentiation of the intimal flap, true lumen (TL), false lumen (FL), aortic branch ostia, and postoperative stent-graft morphology. The entire preprocessing pipeline was implemented as follows: Firstly, raw DICOM sequences were imported and grayscale threshold adjustment was performed to eliminate background noise and enhance vascular tissue contrast. Secondly, the region-growing segmentation algorithm was adopted to extract the vascular region of the aortic arch, and manual fine calibration was conducted to separate the anatomical boundaries of the true and false lumens, avoiding segmentation deviation caused by partial volume artifacts of CT scanning. Thirdly, morphological hole-filling and topological repair operations were carried out to eliminate tiny gaps, broken edges and irregular jagged surfaces of the initial segmented model. Finally, multi-stage surface smoothing was performed to generate high-quality, watertight closed three-dimensional geometric models. This semi-automatic segmentation and reconstruction method ensures the morphological authenticity of patient-specific aortic dissection models and meets the geometric accuracy requirements for subsequent transient CFD numerical simulation, which is consistent with the mature modeling paradigm validated in authoritative aortic hemodynamic research (12). The finalized geometries were exported to ANSYS ICEM CFD 2023 R1 for hybrid meshing (five prismatic layers, y⁺ < 1, with local refinement at stent landing zones and branch ostia).

### 2.5 Numerical setup and boundary conditions (representative CFD case)

Transient pulsatile CFD simulations were performed using ANSYS Fluent 2023 R1 software, with all initial and boundary conditions strictly set in accordance with standardized aortic hemodynamic simulation specifications. For initial flow conditions, the entire fluid computational domain was initialized with zero gauge static pressure and zero initial velocity to ensure uniform starting conditions for all preoperative and postoperative models, eliminating the interference of initial flow field bias on simulation results.

For boundary conditions: (1) the inlet of the ascending aorta was set as a patient-specific pulsatile velocity inlet, with velocity waveforms derived from clinical Doppler ultrasound measurements before and after TEVAR scaled to the patient’s cardiac output; (2) all distal vascular outlets, including the brachiocephalic trunk, left common carotid artery, left subclavian artery and descending aorta, were assigned as three-element Windkessel (RCR) boundary conditions to simulate downstream vascular resistance and compliance, with R₁, R₂, and C iteratively adjusted to match the measured mean arterial pressure (112 mmHg) within ±3%; (3) the aortic wall and postoperative stent-graft surface were defined as rigid no-slip boundaries, which is a classic and reliable simplification strategy for large-diameter aortic CFD simulation.

In terms of blood rheological parameters, blood was regarded as an incompressible fluid following the modified Quemada non-Newtonian viscosity model to accurately capture the shear-thinning hemodynamic behavior in the dissected aortic arch, which can precisely reflect the viscosity changes of blood flow in low-velocity stasis areas of the false lumen. Simulations were run for 5 cardiac cycles with a transient time step size of 0.005 s; data from the final 2 cycles were used for analysis. Convergence was achieved when residuals fell below 1 × 10⁻⁴ and outlet flow waveforms overlapped completely between consecutive cycles. All solver settings, transient iteration schemes, residual convergence criteria, and physical boundary definitions have been fully listed and explained to ensure simulation transparency and reproducibility. This parameter setting scheme is consistent with the latest research on aortic dissection hemodynamic simulation, ensuring the authenticity and accuracy of numerical calculation results.

### 2.6 CFD analysis: rationale and expected findings

To provide a mechanistic interpretation of the hemodynamic impact of the fenestration TEVAR procedure, we established a comparative analytical framework based on established CFD metrics commonly used in aortic dissection hemodynamic studies. The following parameters were selected as quantitative indicators of flow quality and vascular loading:

Streamline morphology: Assessed to evaluate flow disturbance, vortical structures, and branch perfusion patterns.
Systolic pressure gradient: Computed along the aortic centerline to quantify pressure differentials between the true and false lumens.
Wall shear stress (WSS): Calculated as the tangential force exerted by blood flow on the vessel wall, serving as an indicator of hemodynamic stress.
Oscillatory shear index (OSI): Quantified to characterize the directional variability of WSS, with higher values indicating disturbed, multidirectional flow.

**Figure 1:**
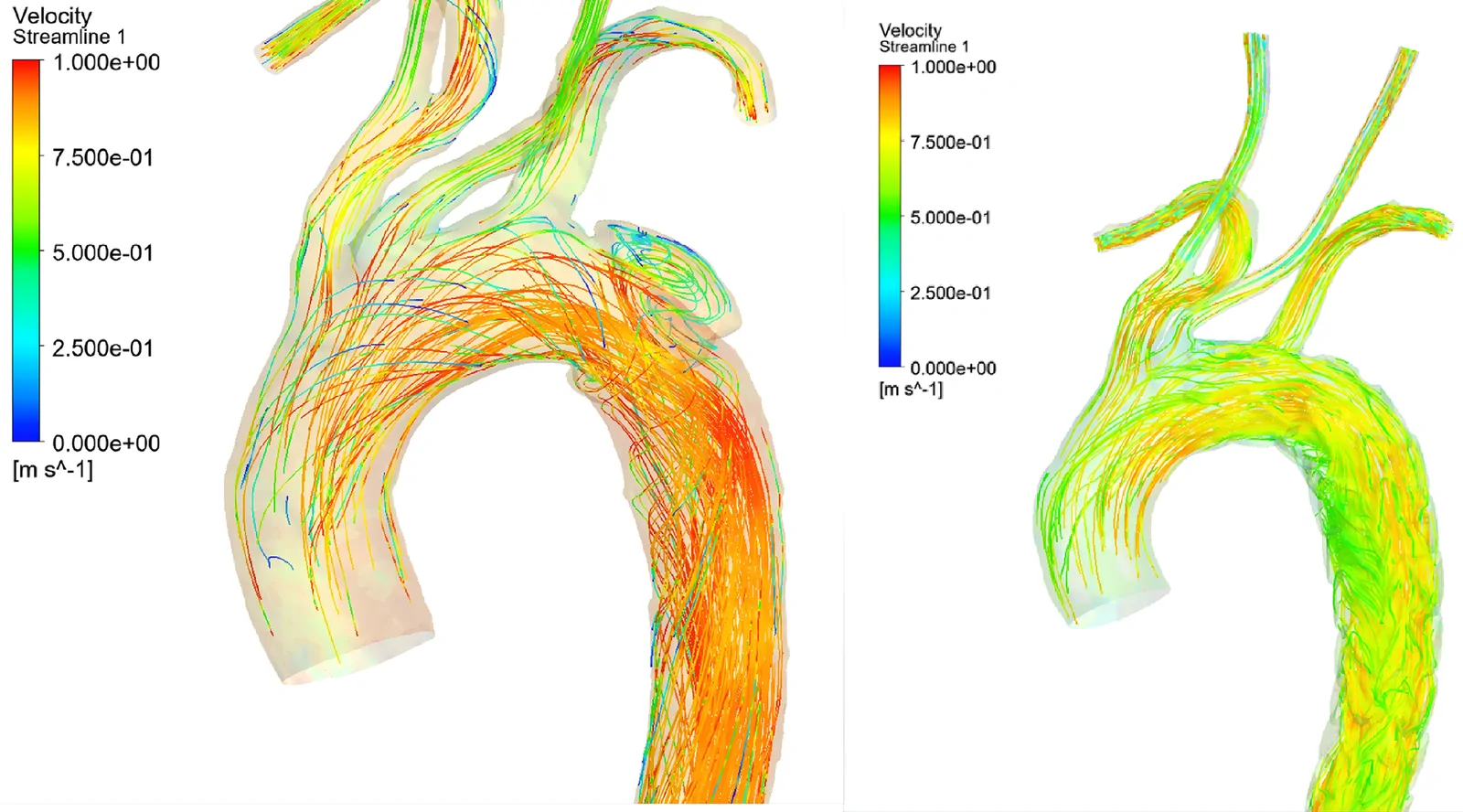
The analysis of streamlined tracking of blood flow velocity field before (left) and after stent-graft placement (right).

**Figure 2:**
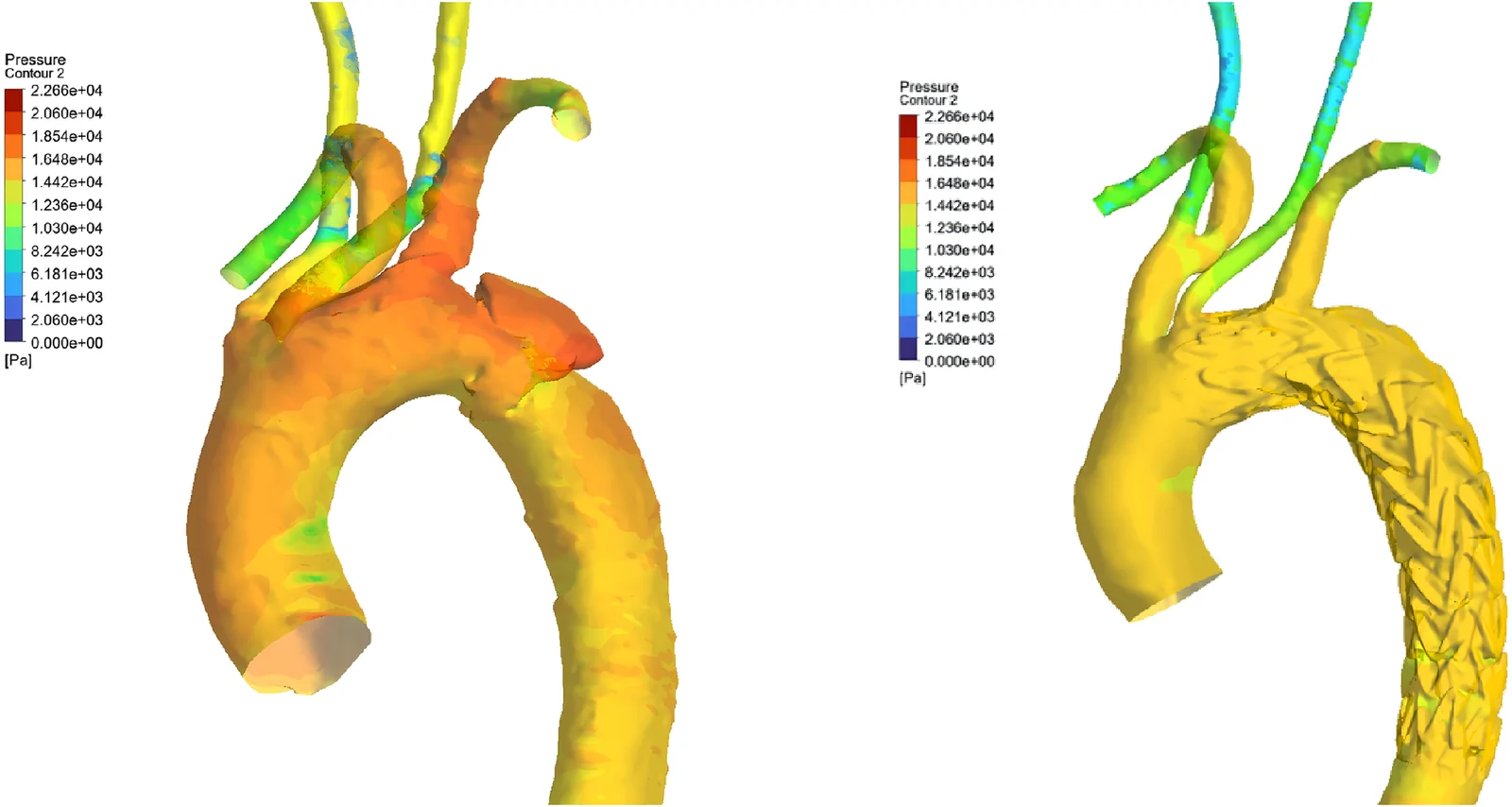
The analysis of pressure before (left) and after stent-graft placement (right).

**Figure 3:**
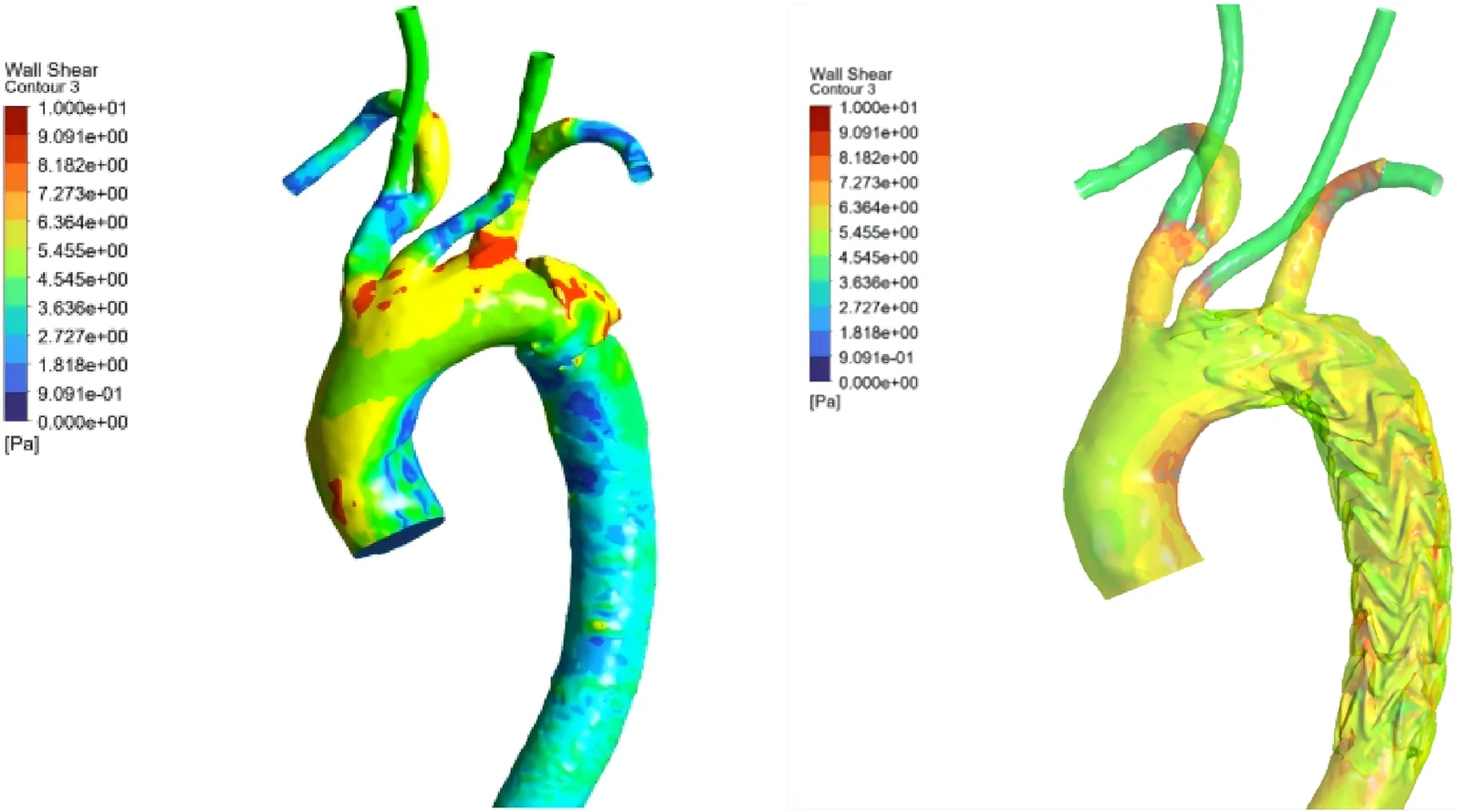
The analysis of wall shear stress on the thoracic aortic wall before (left) and after stent-graft placement (right).

These metrics have been widely adopted in previous CFD investigations of aortic dissection and endovascular repair as objective surrogates for procedural efficacy and aortic remodeling (12). Preoperative and postoperative comparisons of these parameters in the representative case are presented in the Results section (Figures 1–3) and interpreted descriptively.

## 3 Results

### 3.1 Baseline characteristics

Table 1 summarizes a retrospective study of 110 TBAD patients with LSA involvement, mainly males [86 cases (78.2%)], an average age of 58 ± 13 years, and an average BMI of 26.6 ± 4.0 kg/m^2^. Hypertension was common [86 cases (78.2%)], followed by smoking [64 cases (58.2%)]. Other conditions included coronary heart disease [7 cases (6.4%)], chronic lung disease [7 cases (6.4%)], prior stroke [5 cases (4.5%)], diabetes mellitus [5 cases (4.5%)], hyperlipidemia [5 cases (4.5%)], and renal insufficiency [11 cases (10.0%)]. Disease stages were acute [70 cases (63.6%)], subacute [16 cases (14.5%)], and chronic [24 cases (21.8%)]. Preoperative laboratory evaluations revealed a mean serum creatinine concentration of 99.4 ± 114.1 μmol/L and a glomerular filtration rate of 85.0 ± 24.6 mL/min. Computed tomography angiography (CTA) identified critical morphometric parameters: the distance between the LSA ostium and the dissection flap was 1.0 ± 2.8 mm (indicating an inadequate proximal landing zone); the LSA diameter was 9.5 ± 1.3 mm; and the diameter of the ascending aorta at the LSA level was 28.3 ± 2.7 mm. All implanted stent grafts (Medtronic Inc) exhibited a mean diameter of 31.4 ± 2.2 mm, with external fenestrations measuring 8–10 mm to maintain left subclavian artery (LSA) perfusion.

**Table 1:** Demographics of 110 patients.

Variables		Values
Age, years		58 ± 13
Sex	Male	86 (78.2%)
	Female	24 (21.8%)
Body mass index, kg/m^2^		26.6 ± 4.0
Course of disease	Acute	70 (63.6%)
	Subacute	16 (14.5%)
	Chronic	24 (21.8%)
Smoke		64 (58.2%)
Hypertension		86 (78.2%)
Diabetes mellitus		5 (4.5%)
hyperlipidemia		5 (4.5%)
Coronary heart disease		7 (6.4%)
Chronic lung disease		7 (6.4%)
Atrial fibrillation		0 (0%)
Prior stroke		5 (4.5%)
Gastrointestinal hemorrhage		0 (0%)
Recent myocardial infarction		0 (0%)
Renal insufficiency		11 (10.0%)
Tumor history		1 (0.9%)
Previous surgery		10 (9.1%)
Blood test	White blood cell count, 10^6^/L	9.8 ± 4.2
	Serum potassium, mmol/L	3.8 ± 0.5
	Creatinine, umol/L	99.4 ± 114.1
	Glomerular filtration rate, mL/min	85.0 ± 24.6
CTA	The distance between the LSA opening and the interlayer, mm	1.0 ± 2.8
	The diameter of LSA, mm	9.5 ± 1.3
	The diameter of the ascending aorta at LSA, mm	28.3 ± 2.7

### 3.2 Outcomes

The procedures were executed with a 100% technical success rate, and the mean operative duration was 118.7 ± 43.5 min. Early complications included endoleaks: Type I [3 cases (2.7%)], Type II [1 case (0.9%)], and Type IV [3 cases (2.7%)]; LSA stenosis [1 case (0.9%)]; and peripheral vascular complications [2 cases (1.8%)], such as access site hematoma. Mortality was observed in 2 cases [1.8%], both attributed to multiorgan failure unrelated to the procedure. No occurrences of LSA occlusion, vertebral artery ischemia, aortic retrograde tear, pneumothorax, or perioperative myocardial infarction were reported. During follow-up (mean duration 5.7 ± 6.8 months), complete false lumen thrombosis was achieved in 92.7% (102/110) of cases. Delayed complications included LSA occlusion [3 cases (2.7%)], newly developed LSA stenosis [2 cases (1.8%)], and persistent endoleaks: Type I [3 cases (2.7%)], Type II [2 cases (1.8%)], and Type IV [3 cases (2.7%)]. Renal replacement therapy was required in 2 cases [1.8%], persisting from the perioperative period. There were no reports of aortic ruptures or neurological events, such as vertebral artery ischemia. Patients experiencing LSA occlusion and LSA stenosis did not exhibit any overt symptoms of left upper limb ischemia (See Table 2).

**Table 2:** Clinical data during and after the operation.

Variables		Values
Time of operation, min		118.7 ± 43.5
The diameter of the stent, mm		31.4 ± 2.2
endoleak	I	3 (2.7%)
	II	1 (0.9%)
	III	0 (0%)
	IV	3 (2.7%)
Occlusion of LSA		0 (0%)
Stenosis of LSA		1 (0.9%)
Vertebral artery type cerebral ischemia		0 (0%)
Aortic retrograde tear		0 (0%)
Peripheral vascular complications		2 (1.8%)
Pneumothorax		0 (0%)
Dialysis		2 (1.8%)
Death		2(1.8%)

CFD analysis was performed in one representative case (65-year-old male, entry tear 23 mm distal to LSA). Mesh independence was verified by a <2% change in peak systolic velocity upon a 20% increase in element count. Follow-up outcomes are summarized in Table 3.

**Table 3:** Clinical data follow-up.

Variables		Values
Follow-up time, months		5.7 ± 6.8
endoleak	I	3 (2.7%)
	II	2 (1.8%)
	III	0 (0%)
	IV	3 (2.7%)
Occlusion of LSA		3 (2.7%)
Stenosis of LSA		2 (1.8%)
Vertebral artery type cerebral ischemia		0 (0%)
Aortic retrograde tear		0 (0%)
Peripheral vascular complications		0 (0%)
Pneumothorax		0 (0%)
Dialysis		0 (0%)
Death		1(0.9%)

#### 3.2.1 Flow patterns

Pre-operatively, the true lumen showed dense parallel laminar flow with localized streamline compression. A high-velocity jet (1.35 m/s) formed at the entry tear and converged into the false lumen. The LSA exhibited S-shaped distortion, with approximately 30% of systolic flow diverted into the false lumen; the right subclavian artery showed compensatory streamline densification (Figure 1A). Post-operatively, flow converted to a uniform single-lumen laminar pattern. LSA streamlines straightened to an L-shaped configuration, flow volume normalized, and the compensatory changes in the right subclavian artery resolved (Figure 1B).

#### 3.2.2 Pressure

Pre-operatively, peak systolic pressure in the ascending aorta and arch reached 182 mmHg due to true lumen narrowing. Pressure in the descending true lumen dropped to 86–107 mmHg. The false lumen showed a focal high-pressure zone at the jet impaction site (182 mmHg), 10–20 mmHg higher than the true lumen. The right subclavian artery exhibited compensatory hypertension (Figure 2A). Post-operatively, pressure in the stented true lumen normalized to 110–130 mmHg at the arch with a smooth distal decline. LSA perfusion recovered to 110–125 mmHg, and the three arch branch pressures balanced (inter-branch difference <10 mmHg). The false lumen became static and non-pulsatile (Figure 2B).

#### 3.2.3 WSS and OSI

Pre-operatively, WSS peaked at the entry tear edge (30–35 Pa) and at the true lumen stenosis. A focal high-WSS patch (25–30 Pa) was observed at the false lumen impaction site. The LSA ostium showed elevated WSS (25–30 Pa) with an OSI > 0.4. Low-WSS areas (<5 Pa) covered more than 60% of the false lumen surface (Figure 3A). Post-operatively, WSS in the stented segment stabilized at 10–15 Pa; the high-WSS zone at the entry tear disappeared. LSA WSS decreased to 15–20 Pa with an OSI <0.02. False lumen WSS dropped to near-zero (<0.5 Pa) (Figure 3B).

## 4 Discussion

TEVAR is generally the preferred treatment for TBAD; however, it is not recommended for complex cases involving lesions in proximity to major branch arteries or when the stent-attachment area is less than 1.5 cm. Complex TBAD cases present significant challenges, including elevated mortality rates, spinal cord ischemia, and compromised blood supply to branch arteries (6, 7). The primary treatment objectives are to prevent rupture, maintain patency of branch vessels, and ensure adequate organ perfusion. TEVAR is an interventional approach that effectively seals the tear site, preserves the blood supply to the left subclavian artery, and minimizes postoperative complications (10).

Numerous contemporary studies have demonstrated that performing left subclavian artery (LSA) reconstruction during TEVAR for TBAD patients can reduce the incidence of stroke (13, 14). The use of Castor stents (15), the chimney technique (16), and hybrid surgical approaches (17) have all shown favorable outcomes in terms of LSA patency (99.3%/100%/93.3%) and low stroke rates (0%/0%/2%). Similarly, the fenestration technique has demonstrated comparable clinical efficacy. Ye reported that the LSA occlusion rate following fenestration TEVAR was 0%, with an endoleak rate of 4.88% (18). While Wu reported a limb ischemic complication rate of 28.6% following fenestrated TEVAR surgery at their center, which is significantly higher compared to other techniques (19). However, there were only 14 cases, and the small sample size makes the results unreliable.

Our study demonstrated a 100% implantation success rate for the fenestrated TEVAR technique, suggesting its high repeatability and operability. No cases of LSA stenosis or occlusion were observed during the perioperative period, and no strokes occurred. During the follow-up period, the LSA occlusion rate was 2.7%, and the stenosis rate was 1.8%. These outcomes may be attributed to postoperative stent displacement or a mismatch between the fenestration and the LSA, leading to subsequent LSA occlusion or stenosis. Late stenosis may also result from competitive flow or neointimal hyperplasia. Although our cohort was asymptomatic, routine duplex surveillance is recommended. The overall endoleak rate was 7.2%, comprising 2.7% type I endoleak, attributed to suboptimal proximal sealing in calcified arches, 0.9% type II endoleak, and 2.7% type IV endoleak, which is consistent with the self-limiting porosity of Medtronic grafts as reported by the manufacturer [Medtronic VALIANT Navion IFU]. Throughout the follow-up period, there was no alteration in the status of the endoleak. The perioperative mortality rate was recorded at 1.8%, with postoperative multiple organ failure identified as the cause of death. During the follow-up period, the mortality rate was 0.9%, with deaths not attributed to cardiovascular causes. At the time of follow-up, the incidence of complete thrombosis of the false lumen was 92.7%, which is same as the microporous scaffold graft (MSG) technology (20).

The CFD simulation in this representative case offers a mechanistic explanation for the clinical outcomes. The pre-operative false lumen pressure exceeded true lumen pressure by 10–20 mmHg, consistent with the established model that persistent false lumen pressurization drives distal aortic expansion (21, 22). In this representative case, postoperative elimination of this gradient, along with WSS normalization to 10–15 Pa and OSI reduction to <0.02, was observed. Whether these hemodynamic changes directly correlate with the 92.7% complete thrombosis rate observed in the overall cohort remains to be determined in larger studies. These CFD findings are illustrative, not generalizable. The representative case had favorable anatomy (single entry tear, no distal re-entry, adequate landing zone). Whether these hemodynamic changes apply to complex dissections (multiple re-entries, chronic dissections, marginal landing zones) is unknown and requires further study. We view CFD as a hypothesis-generating complement to clinical data, not a standalone predictive tool.

In TBAD patients, due to the tear in the aortic intima, blood flow enters the false lumen and forms pressure. If there is no outlet, the pressure is equal to or even greater than that in the true lumen. Coupled with the gradual thinning of the vessel wall, the risk of aortic rupture is very high. After implanting a stent, its isolation and guiding functions reduce blood flow, thereby reducing the impact of blood flow and the shear stress on the vessel wall, achieving a protective effect (20, 23, 24). Our study further demonstrates that dissection impacts blood flow within the true lumen as well as in the LSA, resulting in a significant pressure differential between the true and false lumens. Regions of elevated WSS are predominantly observed at the stenotic segment of the true lumen and at the tear's margin, thereby heightening the risk of rupture. The application of the fenestrated TEVAR technique not only obliterates the false lumen but also reinstates normal blood flow in the LSA.

## 5 Study limitations

Despite the promising findings, this study has several limitations that should be acknowledged. First, its retrospective and single-center design inherently introduces potential selection bias and limits the generalizability of the results. A prospective, multi-center randomized controlled trial would be required to provide more robust evidence. Second, the relatively short-term follow-up (mean 5.7 months) precludes the assessment of long-term outcomes, such as the durability of the fenestration, long-term stent-graft performance, and the potential for aortic remodeling progression or regression. Long-term surveillance is necessary to confirm the sustained efficacy and safety of this technique. Third, while CFD analysis provides invaluable insights into hemodynamic changes, the simplifications inherent in computational models (e.g., assumptions about vessel wall rigidity and blood properties) may not fully capture the *in vivo* complexity. The results should be interpreted as indicative of trends rather than absolute physiological values. Finally, the study lacks a direct control group comparing external fenestration TEVAR with other LSA revascularization strategies (e.g., chimney grafts, castor single-branched stents, or hybrid surgery). Therefore, comparative efficacy and advantages over established techniques remain to be elucidated in future comparative studies.

## 6 Conclusions

In this single-center retrospective study, the technique of external fenestration TEVAR demonstrated high technical success and favorable early outcomes for the management of TBAD involving the LSA. It effectively promoted false lumen thrombosis and aortic remodeling while preserving LSA perfusion with a low incidence of perioperative neurological and ischemic complications. These hemodynamic findings, while derived from a single illustrative case, suggest a mechanistic rationale for achieving false lumen depressurization through complete entry coverage and an adequate proximal landing zone (≥15 mm when anatomically feasible). However, this observation requires validation in larger prospective studies before being translated into a formal clinical recommendation. Larger prospective studies incorporating CFD into routine pre-procedural planning are needed to validate these mechanistic observations. These findings suggest that external fenestration TEVAR is a viable and effective minimally invasive option for complex TBAD, providing a valuable alternative for extending the proximal landing zone while maintaining branch patency. Further long-term and comparative studies are warranted to solidify its role in the treatment arsenal for aortic dissection.

## Data Availability

The original contributions presented in the study are included in the article/Supplementary Material, further inquiries can be directed to the corresponding author.
